# Estimating the costs of HIV clinic integrated versus non-integrated treatment of pre-cancerous cervical lesions and costs of cervical cancer treatment in Kenya

**DOI:** 10.1371/journal.pone.0217331

**Published:** 2019-06-06

**Authors:** Elisabeth L. Vodicka, Michael H. Chung, Marita R. Zimmermann, Rose J. Kosgei, Fan Lee, Nelly R. Mugo, Timothy C. Okech, Samah R. Sakr, Andy Stergachis, Louis P. Garrison, Joseph B. Babigumira

**Affiliations:** 1 University of Washington, Seattle, Washington, United States of America; 2 University of Nairobi, Nairobi, Kenya; 3 Kenyatta National Hospital, Nairobi, Kenya; 4 Kenya Medical Research Institute, Nairobi, Kenya; 5 United States International University, Nairobi, Kenya; 6 Coptic Hope Center for Infectious Diseases, Nairobi, Kenya; Boston University School of Public Health, UNITED STATES

## Abstract

**Objectives:**

To estimate the modified societal costs of cervical cancer treatment in Kenya; and to compare the modified societal costs of treatment for pre-cancerous cervical lesions integrated into same-day HIV care compared to “non-integrated” treatment when the services are not coordinated on the same day.

**Materials and methods:**

A micro-costing study was conducted at Coptic Hope Center for Infectious Diseases and Kenyatta National Hospital from July 1-October 31, 2014. Interviews were conducted with 54 patients and 23 staff. Direct medical, non-medical (e.g., overhead), and indirect (e.g., time) costs were calculated for colposcopy, cryotherapy, Loop Electrosurgical Excision Procedure (LEEP), and treatment of cancer. All costs are reported in 2017 US dollars.

**Results:**

Patients had a mean age of 41 and daily earnings of $6; travel time to the facility averaged 2.8 hours. From the modified societal perspective, per-procedure costs of colposcopy were $41 (integrated) vs. $91 (non-integrated). Per-procedure costs of cryotherapy were $22 (integrated) vs. $46 (non-integrated), whereas costs of LEEP were $50 (integrated) and $99 (non-integrated). This represents cost savings of $25 for cryotherapy and $50 for colposcopy and LEEP when provided on the same day as an HIV-care visit. Treatment for cervical cancer cost $1,345-$6,514, depending on stage. Facility-based palliative care cost $59/day.

**Conclusions:**

Integrating treatment of pre-cancerous lesions into HIV care is estimated to be cost-saving from a modified societal perspective. These costs can be applied to financial and economic evaluations in Kenya and similar urban settings in other low-income countries.

## Introduction

Cervical cancer is the leading cause of cancer deaths among women in Sub-Saharan Africa (SSA) with an age-standardized mortality rate ranging from 43.3 to 69.8 per 100,000 women [1]. Although preventable, many women in low-income settings arrive at the health facility with pre-cancerous lesions or cervical cancer, requiring time-intensive and invasive treatments that are often prohibitively expensive [2,3]. Women in Kenya, for example, experience an age-standardized cervical cancer mortality rate of 22.8 per 100,000 women and an incidence rate of 33.8 per 100,000 women [4]. Unfortunately, only 3.5% of women aged 25–64 in Kenya have received screening for cervical cancer [5].

The World Health Organization’s recent call to action for the elimination of cervical cancer is generating accelerated opportunities to address cervical cancer incidence and mortality [6]. Providing integrated delivery of cervical cancer screening and treatment for pre-cancerous lesions alongside other services is a promising strategy toward this goal that has been applied in SSA [7–11]. Integrated services leverage the fact that women are already attending a healthcare facility and provide the option to receive cervical cancer prevention services during the same visit. Such strategies have gained support from the WHO and other global organizations, such as Pink Ribbon Red Ribbon which works to simultaneously address HIV and cervical cancer in SSA [6,12,13]. HIV treatment centers are of particular interest for service integration due to concentrated efforts to curb the proliferation of HIV in high prevalence regions and because women living with HIV have a higher incidence of cervical cancer [14,15]. In Kenya alone, approximately 830,000 women age 15 or older were living with HIV in 2015 [16]; among those, nearly 68% of adult women with HIV were on anti-retroviral therapy (ART) for treatment, which commonly requires monthly clinic and/or pharmacy visits for ART prescription renewals [16]. Offering treatment for pre-cancerous lesions to women as they enter the health system for routine HIV care or ART renewal may improve cervical cancer prevention among women living with HIV and may do so at a low societal marginal cost [7,17].

Clinical effectiveness should drive adoption and uptake of appropriate treatment strategies for pre-cancerous lesions and cervical cancer. However, cost analyses are an essential yet often missing component of healthcare planning that can bolster current health policy work by providing an understanding of resource requirements for health service delivery. Prior to scaling up services, locally-specific cost information should be considered [18]. This is particularly true in resource-constrained settings where health care budgets are limited and the external funding environment is uncertain [18]. To our knowledge, existing cost estimates for treating cervical cancer in Kenya are out of date, with most recent estimates from 2005 using data from 2000 [19]. As such, this study assessed costs of integrated and non-integrated treatment for pre-cancerous cervical lesions and updated the costs of cervical cancer treatment in Nairobi, Kenya.

## Materials and methods

We conducted a micro-costing study in 2014 at Coptic Hope Center for Infectious Diseases (CHC) and Kenyatta National Hospital (KNH), Nairobi, Kenya to assess costs of integrated and non-integrated treatment of pre-cancerous lesions from the modified societal perspective. Ethics approval for this study was received from the Kenyatta National Hospital Ethics and Research Committee and the University of Washington Institutional Review Board. Several definitions for “integration” exist, representing different models of care. A recent systematic review of integrating cervical cancer with HIV health care services identified three types of integration, including: 1) “Within-Clinic Integration” where internal staff are relied upon to provide a new set of services complementary to existing services; 2) “Co-Location” which relies upon coordination between co-located clinics and specialists; and 3) Complex programs of integration and coordination [20]. For the purposes of this cost study, we assume integrated care would be provided using Within-Clinic Integration strategies. Provision of pre-cancerous lesion treatment in HIV clinics would rely on internal staff and existing resources to provide care rather than referring women to a separate health facility or to a future (separate) visit date. Under this integration definition, providers would offer cervical cancer prevention services at the time and place where women are already seeking care for HIV treatment (e.g., to pick up monthly antiretrovirals and obtain other related services). In addition to costs of treatment of pre-cancerous lesions, we collected costs of treatment for cervical cancer and cervical cancer palliative care at KNH, though these services are not expected to be integrated. Costs of screening have been reported previously [21].

CHC is a PEPFAR-funded HIV treatment center providing healthcare, including ART and prophylaxis, to HIV-positive individuals. In 2014, it provided services to approximately 8,800 patients over 52,000 visits [22]. CHC also offers preventive services, including cervical cancer screening and treatment. KNH is a public tertiary care center providing preventive and urgent care for individuals across Kenya. KNH has a reproductive health unit actively offering cervical cancer screening and treatment for pre-cancerous lesions and cancer. It also houses a volunteer-based Palliative Care Center that serves patients seeking hospice for late stage diseases, including cervical cancer.

Interviews were conducted with 54 patients receiving treatment for pre-cancerous lesions and cervical cancer at CHC and KNH during the study period (July 1 to October 31, 2014) to provide information on non-medical and indirect costs of care. Patients were referred by providers to the study team; those who agreed to participate were interviewed by researchers in English or Kiswahili, depending on the woman’s preferences, following a structured interview script (see Supplementary Materials). Additionally, 23 providers, lab personnel and administrative staff who were directly engaged in patient care or knowledgeable about direct medical costs participated in interviews. These staff interviews elicited information about typical care patterns, resource use and costs of services. Snowball sampling was used to identify providers that performed treatment for pre-cancerous lesions and cervical cancer, as well as employees involved in accounting, operations, and supplies management [23]. Personnel within laboratories contracted by CHC and KNH were also interviewed. All individuals provided informed consent prior to participation.

Cost estimates included direct medical (e.g., supplies), non-medical (e.g., patient transportation) and indirect costs (e.g., patient time) from the modified societal perspective [24]. Treatment methods included colposcopy-guided biopsy; cryotherapy; LEEP; adverse and serious adverse events associated with precancerous lesions and cancer; treatment strategies for local, regional and distant invasive cancer; and palliative care.

Resource use for each service was based on clinic activities typically used in the detection of and treatment for pre-cancerous lesions at CHC and KNH and cervical cancer at KNH, as identified by interviews. For example, colposcopy-guided biopsy and LEEP were each assumed to require two visits for treatment procedure and results/follow-up, whereas cryotherapy was assumed to require a single visit reflecting local practice. An adverse event was defined to be an event where a patient required one outpatient visit with a clinician whereas a serious adverse event was defined to be an event where a patient required a one-night stay in the hospital. One round trip to the health facility was assumed for adverse events.

Direct medical costs of clinical procedures were derived from patient and staff interviews. These included costs of personnel time, supplies, and lump sum costs incurred by the patient based on published fee schedules set by the facilities (excluding cost categories previously identified). Non-medical costs included facility overhead and patient expenses incurred while seeking care not due to treatment, including patients’ transportation to and from the facility, and meals purchased during care. Indirect costs comprised resources used due to treatment but not directly associated with healthcare services. These included economic costs of lost productivity due to missed work, missing usual non-work activities, and costs of hiring caregivers for children and elderly relatives. Opportunity costs of patients’ time were calculated by applying an average hourly wage derived from patient interviews.

### Estimating costs of HIV clinic integrated vs. non-integrated treatment of pre-cancerous cervical lesions

Costs of treatment of cervical pre-cancerous lesions via colposcopy, cryotherapy, or LEEP were first estimated for a non-integrated scenario. In this scenario, patients were assumed to attend the health care facility for treatment of pre-cancerous lesions only and all required visits would take place on separate days (i.e., treatment visit followed by a subsequent visit to obtain test results). Two additional scenarios were then evaluated to assess costs of integrating treatment into HIV-care centers: 1) A *fully integrated* scenario in which each component of treatment for pre-cancerous lesions was assumed to be conducted on the same day as an HIV-treatment visit (i.e., Visit 1: HIV-related visit + treatment of pre-cancerous lesions; Visit 2: HIV-related visit + treatment results/follow-up); and 2) a *semi-integrated scenario* in which only procedures performed on the first visit were assumed to be conducted on the same day as an HIV treatment visit, while procedures scheduled for subsequent follow-up were to be completed in additional visits to the health center independent of their HIV treatment schedule. We estimated the marginal costs or cost savings to occur when women are able to receive treatment as an add-on service at the time and place of her HIV treatment visit.

**Fig 1:**
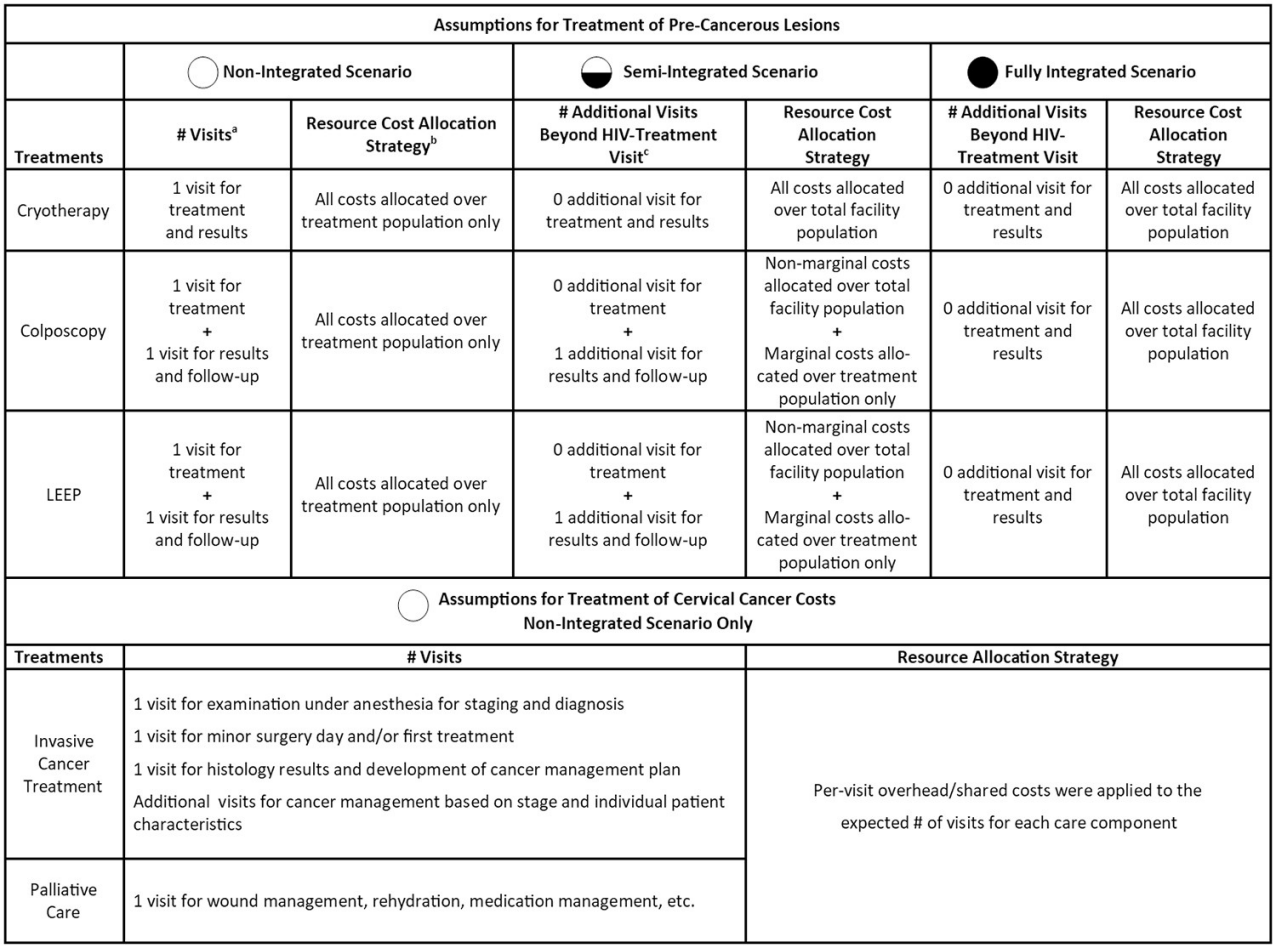
Assumptions for number of visits and allocation of resource costs for treatment of pre-cancerous lesions and cervical cancer under non-integrated and integrated scenarios. ^a^Number of visits refers to the assumed number of times a patient would travel to the clinic for treatment of pre-cancerous lesions under the non-integrated scenario. ^b^Resource costs allocation strategy refers to the method used to allocate costs of shared resources under each scenario. Shared resources included overhead and capital costs, as well as costs of multi-use supplies (i.e., supplies used for both the treatment of pre-cancerous lesions and other non-related services at the HIV-treatment facility). ^c^Number of additional visits beyond HIV-treatment visits refers to the number of times a patient would travel to the clinic for treatment of pre-cancerous lesions under the semi-integrated and fully-integrated scenarios. Zero additional visits for treatment and results assumes that the woman would receive services related to pre-cancerous lesions on the same day as their HIV-treatment visit, creating efficiencies associated with the integration of services. In the semi-integrated scenario, it was assumed that the first pre-cancer visit would be conducted on the same day as an HIV-treatment visit, but any subsequent visits would take place at visits separate from their next HIV-treatment visit. In the fully integrated scenario, it was assumed that all pre-cancer visits would be conducted on the same day as an HIV-treatment visit, leveraging the fact that many women attend HIV-treatment centers at regular intervals for medications and HIV-related care. Additional costing assumptions are provided in Table 1.

Fig 1 further illustrates our assumptions about expected resources used in each integration scenario, taking into consideration potential areas where cost sharing might occur by leveraging existing resources (such as supplies and overhead) while also reducing the travel, time and economic burden for women and their families through within-clinic integration. We anticipated economies of scope would arise from delivering treatment for pre-cancerous lesions jointly with HIV care in an integrated setting. Further, joint delivery of these services may yield new cost efficiencies since costs of shared resources such as overhead would be allocated over multiple service areas rather than cervical cancer prevention programs alone.[25] As such, in the non-integrated setting where services are delivered separately, we assumed that 100% of overhead costs for treatment of cervical precancer would be allocated to the standalone cervical cancer prevention program. When integrating treatment of pre-cancerous lesions as an ancillary service to existing HIV care, we assumed that women would already be attending the facility for HIV-care with treatment for cervical pre-cancer added on to this visit. Therefore, overhead was allocated over all HIV patients in the facility, regardless of whether or not they received the integrated service. Additional details are provided in S1 Table.

**Table 1:** Base case assumptions and calculation methods for societal-level cost estimation.

Cost Component	Assumptions	Source
**Direct Medical Costs**		
Personnel	Personnel costs were calculated using wages based on monthly salaries and estimated proportion of time spent with dedicated to relevant tasks. (Minutes spent with patient)*(Wage per minute) For volunteer-based palliative care, providers’ volunteer time was estimated based on the opportunity cost of their time. (Minutes spent with patient)*(Wage per minute) Wages were based on reported salaries for 2014. Personnel costs included staff training, which was a combination of annual fixed training costs and staff practicum costs (per-minute wage rate multiplied by number of minutes spent in training).	Clinical and administrative interviews
Supplies	Assumed all reusable supplies had a 5-year lifespan at 2014 level of utilization; costs were annuitized at 3%. Assumed reusable supplies specific to cervical cancer screening had a 5-year lifespan at current annual level of screenings, whereas reusable non-specific supplies were allocated over the total number of unique patients receiving care at CHC; costs were annuitized at 3%.	Administrative and clinical interviews; CHC and KNH financial reports; estimates from literature, as needed.
Other Direct Medical Costs for Clinical Procedures	Other direct medical costs for clinical procedures included additional costs incurred by patients beyond the costs of the personnel time and supplies identified through micro-costing. These included additional out of pocket costs/charges to patients per procedure (consultation fees, set hospital charges). Hospital charges to patients included a consultation fee (USD $6.51), plus a fixed fee for services that were set and published by the hospital.	Clinical and administrative interviews
Lab Costs for Treatment of Pre-Cancerous Lesions	Histology costs included laboratory supplies and laboratory staff costs, including recording and preparing slides to be sent for processing, reading slides, quality control and re-reads. Estimated time for lab staff to conduct histology was 2.5 minutes of lab tech time and 10 minutes of pathologist’s time per slide. We assumed one re-read for all laboratory procedures.	Laboratory interviews
Staging and Lab Costs for Cervical Cancer	Staging included the average charge to patients for staging and diagnosis or in the hospital as determined by patient interviews. The average lab costs for advanced treatment were not micro-costed as part of the study, so we applied the weighted average cost of lab services for treatment of precancerous lesions and adverse events to account for utilization.	Patient and laboratory interviews
Direct Medical Costs for Cancer Treatment and Palliative Care	Direct medical costs of cervical cancer treatment were derived from published facility fee schedules and administrative interviews. Services included in the base case scenario represent the most commonly performed services for each stage.	Administrative interviews
**Non-Medical Costs**		
Patient Transport	Patient transport costs were estimated using the average travel time obtained through interviews multiplied by the average wage per minute, plus bus fare or gas.	Patient interviews
Meals for Patient and Visitors	Meal costs included items purchased by the patient or visitors over the course of patient’s treatment.	Patient interviews
Overhead	Integrated Costs of Pre-Cancerous Lesions: Assumed costs of shared resources like overhead would be allocated over the entire clinic population, leveraging existing infrastructure of HIV treatment centers. Therefore, integrated overhead costs are allocated over the total clinic population, resulting in a lower per-visit estimate. Non-Integrated Costs of Pre-Cancerous Lesions: Assumed non-integrated costs would be allocated to the screening and pre-cancerous lesion patient population, resulting in a higher per-visit estimate. Costs for Treatment of Cervical Cancer: Overhead costs for these services are presented as the cost of total overhead over the total number of visits annually to reflect the overhead visit cost per visit for cervical cancer treatment.	Administrative interviews
**Indirect Costs**		
Productivity losses	Productivity losses includes loss of earnings for missed work and opportunity costs of missing other usual activities for the patient and any visitors that accompanied the patient to the facility. Lost earnings were estimated by applying the number of minutes missed from work and usual activities to the average wage per minute. Average hourly wage (further converted to wage per minute) was based on daily wages identified through the patient questionnaire. We assumed 260 working days per year and 8-hour work days.	Patient Interviews
Child/Elderly Care	Weighted average of expenses incurred from hiring a caregiver for children or elderly family members while seeking care. Expenses were identified through the patient interviews.	Patient Interviews

### Updating cost estimates for treatment of cervical cancer in Kenya

In addition to estimating the costs of integrated and non-integrated treatment for pre-cancerous lesions, this study provides cost estimates for cervical cancer treatment by stage as an update to those in the literature. Cervical cancer treatment is only provided at KNH and is not expected to be integrated into HIV care, thus, we present only one set of cost estimates here. While individual treatment plans may vary, we developed base case treatment scenarios with expected resource utilization at each stage based on local treatment regimens identified through interviews and clinical guidelines from the International Federation of Gynecology and Obstetrics (FIGO) [26]. Local invasive cancer treatment included consultation, radical or simple hysterectomy, pain-relief medications (e.g., morphine), and 4 post-treatment follow-up visits. Regional invasive cancer treatment included consultation, radical hysterectomy, 28 sessions of radiotherapy, 3 sessions of chemotherapy, pain-relief medications, and 4 post-treatment follow-up visits. Finally, distant invasive cancer treatment included consultation, 28 sessions of radiotherapy, 3 sessions of chemotherapy, pain-relief medications, and 4 post-treatment follow-up visits.

For all stages of care, we included direct medical, non-medical and indirect costs for three visits: 1) staging and diagnosis; 2) test results and cancer management planning; 3) first treatment or surgery. For patients receiving radiotherapy and/or chemotherapy, we did not collect data on whether they stayed overnight in the hospital, paid for accommodations or meals for the duration of their treatment, or required transportation to KNH every day for the course of treatment. As such, for simplification we did not estimate non-medical and indirect costs incurred during each session of radiotherapy or chemotherapy.

Follow-up visits were expected to cost the same as a cytology visit, including speculum exam and cytological smear,[27] and were based on previously reported costs from CHC and KNH.[21] Palliative care visits at KNH are typically provided by volunteer providers, last 60 minutes, and include the following services: consultation, family therapy, wound dressing, renewal of prescriptions, and rehydration. Opportunity costs of a provider’s time were estimated by applying the average wage for relevant clinical staff weighted by the proportion of time the staff member was expected to spend volunteering on palliative care tasks.

### Base case and uncertainty analysis

Cost estimates for delivering treatment for pre-cancerous lesions and cervical cancer were derived from staff interviews, in addition to published KNH fee schedules when available. Mean cost estimates were used for all base case treatment scenarios. To evaluate components of care that were influential on total costs, we conducted one-way sensitivity analyses where each parameter was varied individually using high and low values equivalent to +/- 20% of base case estimates. A Monte Carlo simulation was performed over 10,000 iterations for each cost model parameter to establish 95% credible ranges around mean base case cost estimates using a normal distribution for all inputs. Scenario analyses were also conducted to evaluate potential variation in treatment costs based on different treatment strategies, as identified by FIGO.

All costs were collected in 2014 Kenyan Shillings and inflated to 2017 Kenyan Shillings using annual Consumer Price Indices for appropriate years [28]. Kenyan Shillings were then converted to 2017 U.S. dollars using official World Bank exchange rates [29].

## Results

We conducted interviews with 23 administrative, clinical and laboratory staff, as well as 54 women attending CHC and KNH for treatment of precancerous lesions and cervical cancer. Patients who participated in the study reported a mean age of 41 and a daily wage of $6. Two-thirds had completed at least secondary school. The vast majority of patients (93%) commuted to the health facility by bus with the average trip taking 2.8 hours one-way. Weighted average transportation costs were approximately $7 each way. Nearly 70% missed an average of eight hours of work to attend the facility and 46% missed an additional 2 hours of non-work activities. This represents approximately $6 of productivity loss for patients and others accompanying them to the facility. Women also reported spending a weighted average $2 per visit on caregiver costs and meals. (Table 2)

**Table 2:** Characteristics of patients attending CHC and KNH for treatment of pre-cancerous lesions and cervical cancer (N = 54) †.

Mean Age	41 years
**Education Level**	
% Less than primary school (<8 years)	11%
% Primary school (8 years)	22%
% Secondary school or vocational training (8–12 years)	56%
% University or higher (>12 years)	11%
**Average Daily Wage (8-hour work day)**	$6.26
**Mode of Transportation to Facility**	
Walk	1.85%
Bus	92.59%
Car	3.70%
Other	1.86%
**Average Travel Time to Facility, One-Way**	2.83 hours
**Average Transportation Costs to Facility, One-Way**^††^	$6.75
**Average Cost of Meals Purchased During Care, Per Visit**^†††^	$1.01
**Average Cost of Hiring Caregiver to Look after Child or Elderly Family Member While Seeking Care, Per Visit**	$1.34
**Opportunity Costs for Patients and Others Involved in Care, Per Visit**	
% of patients who missed work	69%
% of patients who missed usual activities outside of work	46%
% of patients accompanied to visit by someone else	19%
% of visitors who missed work to accompany patient	100%
% of visitors assumed to miss usual activities outside of work	0%
Average amount of work missed (among those who missed)	8.05 hours
Average amount of time missed of usual activities outside of work	2.19 hours
Weighted costs for patients who missed work and usual activities outside of work	$5.11
Weighted costs of missed work/usual activities for visitors accompanying patient	$1.17

^†^All costs are reported in 2017 USD.

^††^For patients who reported driving a car as their mode of transportation to the facility, cost of fuel per liter was estimated at $1.07 with mileage estimated at 0.07 liters per kilometer.

^†††^Meal costs include costs of meals purchased by woman seeking treatment, as well as any meals purchased by visitors accompanying her to the facility.

**Fig 2:**
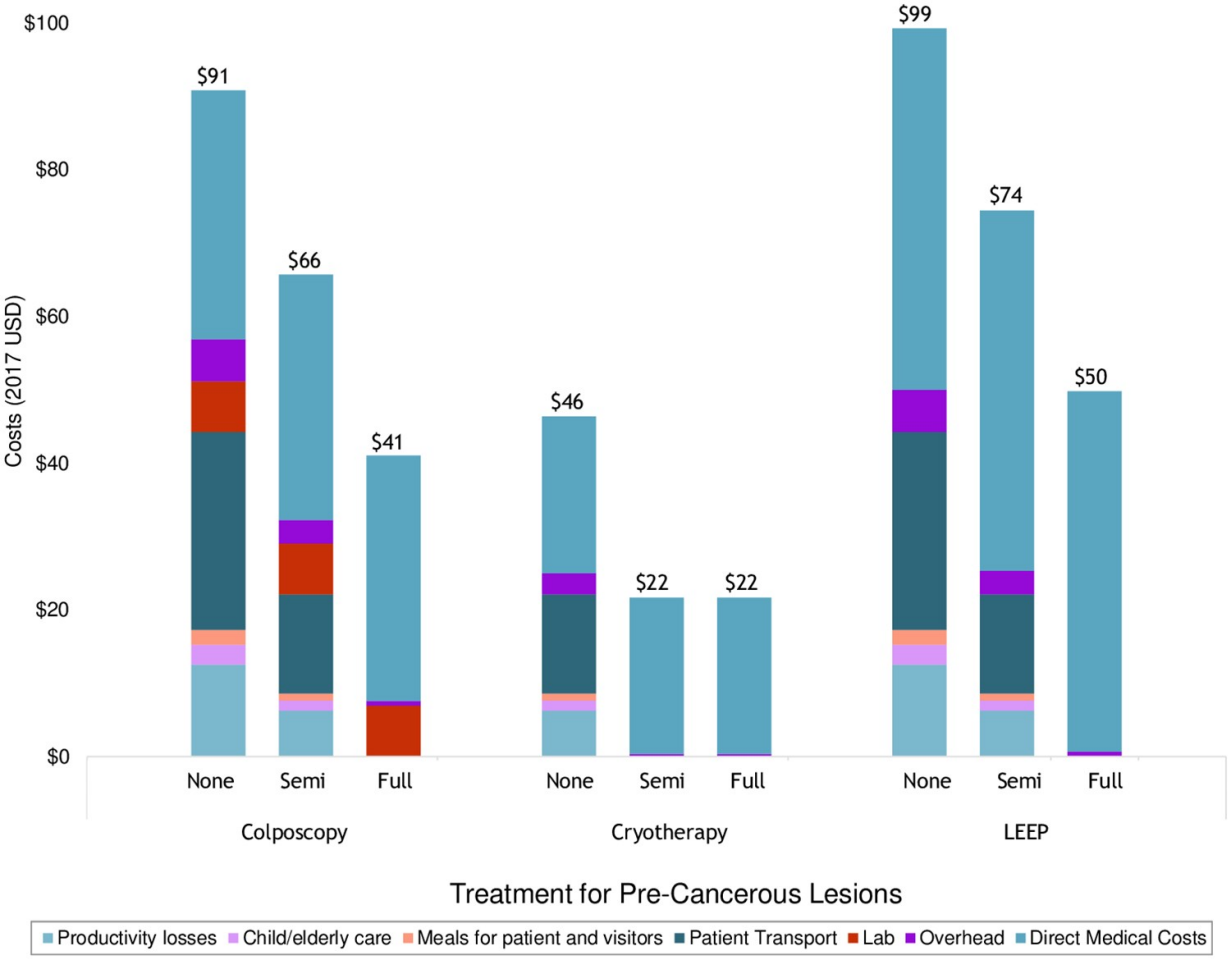
Comparison of integrated vs. non-integrated costs of treatment for pre-cancerous lesions by care component.

Under the base case assumptions of a non-integrated scenario from the modified societal perspective, total per-procedure costs for treating pre-cancerous lesions were $91 for colposcopy, $99 for LEEP and $46 for cryotherapy (Table 3). Integrated per-procedure costs were lower for each treatment. In the semi-integrated scenario, colposcopy was $66, LEEP was $74, and cryotherapy was $22, representing respective cost reductions of 28%, 25%, and 53% compared to non-integrated treatment (Fig 2). In the fully-integrated scenario, colposcopy was $41, LEEP was $50, and cryotherapy was $22, representing respective cost reductions of 55%, 50% and 53% compared to non-integrated treatment.

**Table 3:** Non-integrated and integrated costs to society for treatment of pre-cancerous lesions integrated into HIV-Care (2017 USD).

	Colposcopy			Cryotherapy			LEEP		
Costs	Non-Integrated	Semi-Integrated	Fully Integrated	Non-Integrated	Semi-Integrated	Fully Integrated	Non-Integrated	Semi-Integrated	Fully Integrated
	(95% CI)	(95% CI)	(95% CI)	(95% CI)	(95% CI)	(95% CI)	(95% CI)	(95% CI)	(95% CI)
Direct medical costs for clinical procedure	33.92 (27.00, 40.79)	33.45 (26.60, 40.22)	33.45 (26.60, 40.22)	21.40 (17.07, 25.63)	21.33 (16.98, 25.55)	21.33 (16.98, 25.55)	49.31 (39.36, 59.33)	49.13 (39.25, 59.11)	49.13 (39.25, 59.11)
*Personnel time*	0.65 (0.52, 0.78)	0.65 (0.52, 0.78)	0.65 (0.52, 0.78)	0.19 (0.15, 0.22)	0.19 (0.15, 0.22)	0.19 (0.15, 0.22)	1.10 (0.88, 1.32)	1.10 (0.88, 1.32)	1.10 (0.88, 1.32)
*Supplies*	2.98 (2.40, 3.58)	2.51 (2.00, 3.01)	2.51 (2.00, 3.01)	13.72 (10.96, 16.44)	13.65 (10.87, 16.36)	13.65 (10.87, 16.36)	10.39 (8.27, 12.48)	10.21 (8.16, 12.26)	10.21 (8.16, 12.26)
*Other direct medical costs incurred by patients*	30.29 (24.08, 36.43)	30.29 (24.08, 36.43)	30.29 (24.08, 36.43)	7.49 (5.96, 8.97)	7.49 (5.96, 8.97)	7.49 (5.96, 8.97)	37.82 (30.21, 45.53)	37.82 (30.21, 45.53)	37.82 (30.21, 45.53)
Lab costs	6.93 (5.54, 8.33)	6.93 (5.54, 8.33)	6.93 (5.54, 8.33)	0.00 (0.00, 0.00)	0.00 (0.00, 0.00)	0.00 (0.00, 0.00)	0.00 (0.00, 0.00)	0.00 (0.00, 0.00)	0.00 (0.00, 0.00)
Overhead	5.74 (4.61, 6.87)	3.20 (2.56, 3.85)	0.67 (0.54, 0.81)	2.87 (2.28, 3.44	0.34 (0.27, 0.40)	0.34 (0.27, 0.40)	5.74 (4.62, 6.92)	3.20 (2.57, 3.84)	0.67 (0.53, 0.80)
Patient Transport	27.00 (21.77, 32.41)	13.50 (10.72, 16.19)	0.00 (0.00, 0.00)	13.50 (10.80, 16.21)	0.00 (0.00, 0.00)	0.00 (0.00, 0.00)	27.00 (21.265, 32.54)	13.50 (10.81, 16.26)	0.00 (0.00, 0.00)
Meals for patient and visitors	2.02 (1.63, 2.42)	1.01 (0.81, 1.21)	0.00 (0.00, 0.00)	1.01 (0.81, 1.21))	0.00 (0.00, 0.00)	0.00 (0.00, 0.00)	2.02 ((1.62, 2.43)	1.01 (0.81, 1.21)	0.00 (0.00, 0.00)
Child/elderly care	2.68 (2.13, 3.20)	1.34 (1.07, 1.61)	0.00 (0.00, 0.00)	1.34 (1.08, 1.62)	0.00 (0.00, 0.00)	0.00 (0.00, 0.00)	2.68 (2.15, 3.21)	1.34 (1.07, 1.61)	0.00 (0.00, 0.00)
Productivity losses*	12.55 (10.03, 15.06)	6.28 (5.02, 7.57)	0.00 (0.00, 0.00)	6.28 (5.01, 7.52)	0.00 (0.00, 0.00)	0.00 (0.00, 0.00)	12.55 (10.04, 15.06)	6.28 (5.04, 7.53)	0.00 (0.00, 0.00)
**Total**	**90.84** **(72.71, 109.08)**	**65.71** **(52.32, 78.98)**	**41.05** **(32.68, 49.36)**	**46.40** **(37.05, 55.63)**	**21.67** **(17.25, 25.95)**	**21.67** **(17.25, 25.95)**	**99.30** **(79.44, 119.49)**	**74.46** **(59.55, 89.56)**	**49.80** **(39.78, 59.91)**

*Productivity losses include loss of earnings from missed work and opportunity costs of missing other usual activities. Estimates include losses for both the patient and any visitors that accompanied the patient to the facility for care.

Treatment of adverse and serious adverse events cost $109 and $790, respectively (Table 4). Total costs for a full course of treatment was $1,345 for local invasive cancer, $6,514 for regional invasive cancer, and $5,152 for distant invasive cancer, with direct medical costs comprising the majority of the total. Direct medical costs for staging and lab services were estimated to be $105. Simple hysterectomy was $887 vs. $1,361 for radical hysterectomy. Each session of radiotherapy and chemotherapy cost $142 and $236, respectively, and four follow-up visits were estimated at $159. When incurred, hospital consultation fees were $7 and medications for pain relief were $118. Finally, each day of facility-based palliative care was estimated to cost $59, including the following direct medical costs: palliative consultation ($4), family therapy ($7), wound dressing ($7), renewal of prescriptions ($2), and rehydration services ($18). Non-medical and indirect costs for each treatment strategy ranged from $22-$76.

**Table 4:** Estimated costs to society of treatment of cervical cancer in Nairobi Kenya (2017 USD)*.

Treatment Strategies	Costs	95% Credible Range
***Treatment for Adverse Events***		
Direct medical costs for clinical procedure	$79.14	($63.35, $94.53)
Personnel	$72.11	($57.73, $86.10)
Supplies	$7.03	($5.62, $8.43)
Other direct medical costs incurred by patients	$0.00	($0.00, $0.00)
Lab costs	$6.93	($5.54, $8.34)
Overhead	$0.31	($0.25, $0.38)
Patient transport	$13.50	($10.79, $16.22)
Meals for patient and visitors	$1.01	($0.81, $1.21)
Child/elderly care	$1.34	($1.07, $1.61)
Productivity losses**	$6.28	($5.01, $7.55)
**Treatment of Adverse Events, Total Cost**	**$108.51**	**($86.82, $129.84)**
***Treatment for Serious Adverse Events***		
Direct medical costs for clinical procedure	$761.06	($612.06, $916.07)
Personnel	$754.03	($606.44, $907.64)
Supplies	$7.03	($5.62, $8.43)
Other direct medical costs incurred by patients	$0.00	($0.00, $0.00)
Lab costs	$6.93	($5.54, $8.34)
Overhead	$0.31	($0.25, $0.38)
Patient transport	$13.50	($10.79, $16.22)
Meals for patient and visitors	$1.01	($0.81, $1.21)
Child/elderly care	$1.34	($1.07, $1.61)
Productivity losses**	$6.28	($5.01, $7.55)
**Serious Adverse Events Total**	**$790.43**	**($635.53, $951.38)**
***Local Invasive Cancer***		
Staging and lab costs	$105.38	($83.67, $126.73)
Direct medical costs for treatment	$1,012.67	($809.16, $1,212.78)
Consultation	$6.51	($5.20, $7.81)
Simple hysterectomy	$887.79	($709.39, $1,063.25)
Medications for pain relief (e.g., morphine)	$118.37	($94.57, $141.72)
Overhead	$2.51	($1.99, $3.02)
Patient transport	$40.49	($32.50, $48.64)
Meals for patient and visitors	$3.03	($2.43, $3.65)
Child/elderly care	$4.02	($3.22, $4.83)
Productivity losses**	$18.83	($15.05, $22.64)
Four Post-Treatment Follow-Up Visits	$158.54	($126.41, $190.16)
**Local Invasive Cancer Treatment Total**	**$1,345.47**	**($1,074.43, $1,612.45)**
***Regional Invasive Cancer***		
Staging and lab costs	$105.38	($83.67, $126.73)
Costs for treatment	$6,173.68	($4,956.44, $7,418.39)
Consultation	$6.51	($5.20, $7.81)
Radical hysterectomy	$1,361.27	($1,087.09, $1,633.38)
Radiotherapy, cost for 28 sessions	$3,977.29	($3,198.71, $4,780.96)
Chemotherapy, cost for 3 sessions	$710.23	($570.87, $854.52)
Medications for pain relief (e.g., morphine)	$118.37	($94.57, $141.72)
Overhead	$9.73	($7.70, $11.69)
Patient transport	$40.49	($32.50, $48.64)
Meals for patient and visitors	$3.03	($2.43, $3.65)
Child/elderly care	$4.02	($3.22, $4.83)
Productivity losses**	$18.83	($15.05, $22.64)
Four Post-Treatment Follow-Up Visits	$158.54	($126.41, $190.16)
**Regional Invasive Cancer Total**	**$6,513.69**	**($5,227.42, $7,826.73)**
***Distant Invasive Cancer***		
Staging and lab costs	$105.38	($83.67, $126.73)
Costs for treatment	$4,812.40	($3,866.93, $5,768.01)
Consultation	$6.51	($5.20, $7.81)
Radiotherapy, cost for 28 sessions	$3,977.29	($3,197.34, $4,768.12)
Chemotherapy, cost for 3 sessions	$710.23	($569.82, $850.36)
Medications for pain relief (e.g., morphine)	$118.37	($94.57, $141.72)
Overhead	$9.73	($7.70, $11.69)
Patient transport	$40.49	($32.50, $48.64)
Meals for patient and visitors	$3.03	($2.43, $3.65)
Child/elderly care	$4.02	($3.22, $4.83)
Productivity losses**	$18.83	($15.05, $22.64)
Four Post-Treatment Follow-Up Visits	$158.54	($126.41, $190.16)
**Distant Invasive Cancer Total**	**$5,152.41**	**($4,137.91, $6,176.35)**
***Palliative Care Visit***		
Direct medical costs for clinical procedure	$36.70	($32.57, $40.83)
Palliative consultation	$3.55	($2.84, $4.27)
Family therapy	$6.51	($5.23, $7.78)
Wound dressing	$6.51	($5.21, $7.85)
Renewal of prescriptions	$2.37	($1.91, $2.84)
Rehydration	$17.76	($14.24, $21.37)
Overhead	$0.31	($0.25, $0.38)
Patient transport	$13.50	($10.79, $16.22)
Meals for patient and visitors	$1.01	($0.81, $1.21)
Child/elderly care	$1.34	($1.07, $1.61)
Productivity losses**	$6.28	($5.01, $7.55)
**Palliative Care Visit Total**	**$59.13**	**($47.36, $71.08)**

*Costs of cervical cancer treatment are not expected to be integrated into HIV-care; thus, all advanced treatment costs presented are non-integrated.

**Productivity losses include lost earnings from missed work and opportunity costs of missing other usual activities for the patient and any visitors.

Varying each individual parameter by +/- 20% through the one-way sensitivity analysis resulted in potential increases/decreases in total societal costs of treatment ranging in magnitude from 0–20%. The potential impact of parameter uncertainty varied across strategies. Non-integrated costs of pre-cancerous lesion treatment were most sensitive to changes in costs of patient transportation, productivity losses, and overhead for cryotherapy and LEEP, and lab/direct medical costs for colposcopy. Patient transportation, productivity loss and overhead costs became less influential in the semi-integrated and fully-integrated scenarios. For example, productivity losses for colposcopy, cryotherapy and LEEP in the non-integrated scenario were $12.55, $6.28, and $12.55, respectively. However, when these treatments were assumed to occur on the same day as an HIV-treatment visit, reducing the number of visits and length of time that a woman would need to spend seeking care, expected productivity losses were reduced by 50–100% and no longer influential on total costs. Similarly, in a non-integrated scenario, patient transportation costs were $27 for two round-trip visits for colposcopy and LEEP, and $13.50 for one round-trip visit for cryotherapy. In contrast, in a fully-integrated scenario, $0 in marginal transportation costs were expected for all treatment methods.

When evaluating costs of different stage-based cancer treatment strategies to reflect variation in care (S2 and S3 Tables), direct medical costs of treatment options for local invasive cancer ranged from $1,277 for treatment with simple hysterectomy, to $4,366 for treatment with radiotherapy when surgery was contraindicated, to $5,254 for treatment with simple hysterectomy followed by radiotherapy (S4 Table). Direct medical costs for regional invasive cancer treatment ranged from $4,366 for treatment with radiotherapy only, to $5,076 for chemotherapy and radiotherapy combined, to $6,438 for radical hysterectomy, chemotherapy and radiotherapy. Finally, direct medical costs for distant invasive cancer treatment ranged from $5,076 for chemotherapy and radiotherapy to $5,113 for chemotherapy, radiotherapy, plus a day of facility-based palliative care. Adding palliative care to any treatment scenarios would add $37 in direct medical costs per day.

## Discussion

Our study evaluated costs of integrating treatment for pre-cancerous lesions into an HIV-treatment center in Kenya and demonstrated that, from a modified societal perspective, integrated care can provide substantial cost savings—on the order of 25–55%. We estimated that savings from integrated services compared to non-integrated services would come from lower marginal costs of patient transportation, indirect costs, shared costs of overhead and reusable supplies (i.e., specula and standard clinical sterilization supplies). Given that many savings occur at the patient-level, integration holds potential to reduce the financial and economic burden incurred by women who require treatment. However, costs should be combined with effectiveness data to assess the value achieved due to integration.

Cost and cost-effectiveness estimates of integrated health services have been identified as important research gaps in global health [18]. However, few cost estimates for integrated treatment of pre-cancerous lesions are available in the literature. To our knowledge, this is the first study to assess costs of treatment for pre-cancerous lesions integrated into HIV care in Kenya. A 2013 study evaluated the costs and cost-effectiveness of integrating cervical cancer screening into care for HIV-positive women in South Africa and estimated a lower cost per procedure for colposcopy ($68–75; range: $51-$94[2013 USD]]) than our findings from Kenya ($91; 95% CR: $73-$109) [17]. Cost differences between our findings and the South African estimates are likely due to the different perspectives applied for the analyses. The South Africa study was conducted from a provider perspective, which did not include non-medical or indirect costs [17]. In contrast, Kenya costs were estimated from the modified societal perspective and included opportunity costs of patients’ time.

Cost differences may also reflect variations in underlying assumptions, methods for cost calculations, and data availability for each study. While efforts are underway to create standardized guidelines for costing studies in low- and middle-income settings [25], more guidance is needed on costing methods for integrated services, particularly for shared costs such as overhead. For example, we allocated the marginal overhead costs of integrated services for the treatment of pre-cancerous lesions over the total patient population at the HIV clinic. This may lead to an underestimate of integrated overhead cost. Future research should focus on rigorously assessing the methods of measuring and calculating variables likely to change under integration, such as overhead and potential for newly integrated services to displace existing services.

This study also updated cost estimates of treatment for cervical cancer, including facility-based palliative care. Few estimates of cervical cancer treatment costs are available for comparison in Sub-Saharan Africa. To our knowledge, most recently published costs of cervical cancer treatment in Kenya are based on estimates published in 2005 using data from 2000 [19]. These estimates ranged from $1,383 for treatment for local invasive cancer with radical or simple hysterectomy to $1,923 for treatment with radiotherapy for regional and distant invasive cancer (2000 International $) [19]. Similar estimates were derived for Tanzania using average direct medical cost data from 2002–2011 with cervical cancer treatment costs ranging from $1,741 to $2,989 (2013 USD), depending on stage [30]. Our estimates ranged from $1,345 to $6,514 for cancer treatment and reflect combined use of hysterectomy, radiotherapy, chemotherapy and palliative care based on standard KNH practices at the time of the study.

Published cost estimates for cervical cancer palliative care are limited. We estimated that facility-based cervical cancer palliation would cost $59 per day from the modified societal perspective. In comparison, one study in South Africa identified general hospital-based palliative care to cost $80 per visit (2007 USD) from a provider perspective [31]. Of note, the study incorporated higher capital costs and did not include patient-level costs. For women who arrive to health care facilities with late stage cervical cancer, standard treatment may not be an option [6]. In these cases, palliation can reduce pain and improve quality of life over the duration of her illness. Combined with health and quality of life impact, our palliative care costs can be used to understand the potential economic impact of strengthening and scaling up cervical cancer palliative care programs.

Our study has several limitations. Cost estimates derived through this study reflect standard practices in Nairobi and may not generalize to costs in rural areas. While many women from outside of Nairobi seek treatment at KNH (traveling an average of 2.8 hours each way–and up to 12 hours each way–in our study), women with resources to travel to KNH may be different in terms of health-seeking behavior, average wage, and other factors compared to women who require treatment but do not attend the facility. Additionally, cancer treatment costs were based on the most commonly reported treatment regimen at a public tertiary hospital at the time of the study. However, on-the-ground treatment decisions may vary based on cancer stage, individual patient characteristics and preferences, health facility resource availability, supply chain factors, provider preferences and access to training, strength of health systems, and facility type. This variation in treatment strategies challenges the strength of the base case cost estimate of a typical course of treatment. As such, a range of treatment regimens were assessed in order to identify costs that may be tailored to a specific patient or practice (S4 Table). Additionally, our analysis was conducted from the modified societal perspective and included patient-level non-medical and indirect costs incurred while seeking care; however, the burden of cervical cancer may have a broader impact due to other productivity losses from premature illness and time in convalescence not captured.

Furthermore, while the study examined costs and resource use for facility-based palliative care, it did not assess the costs of home- or institution-based palliative care, which many patients may prefer to in-hospital end-of-life care. Also, facility-based palliative care may not be readily available to patients seeking treatment for cervical cancer. At KNH, for example, palliative care providers work as volunteers on top of their other duties. Therefore, palliative care costs may vary based on availability of services, average wages of the personnel volunteering, and consistent operation of the palliative care center. In general, better data on the value of palliative care is needed. Future studies might evaluate and compare potential health benefits of palliative care when consistently available, training curriculum for palliative care provision, comparisons of palliative care staffing models (e.g., volunteer vs. paid staff, task-shifting, community health worker models, etc.), and home-based palliative care.

It is important to note that while integrating treatment for pre-cancerous lesions as a new service line in HIV-treatment centers could create efficiencies of scope, doing so may also create new challenges for the health system and patient access to care. Integrating services could produce strains on staffing, room availability and funding allocation, thereby necessitating new investments that could limit expected cost savings of integrated services. This study did not assess potential costs associated with new staff, equipment or office space that may be necessary to effectively integrate services for HIV care and treatment of pre-cancerous lesions.

However, despite the limitations, this study provides a comparison of costs for treatment of pre-cancerous lesions integrated and non-integrated strategies in Kenya, in addition to updating published cervical cancer cost estimates. Results from this analysis may be combined with effectiveness data in integrated and non-integrated settings to evaluate the potential cost-effectiveness and budget impact of local and national cervical cancer treatment programs.

## Conclusions

Integrating treatment of pre-cancerous lesions into HIV care is estimated to achieve between a 25% and 55% reduction in costs compared to non-integrated treatment from a modified societal perspective. Cost savings are expected primarily due to reductions in per-patient non-medical costs, such as overhead, and indirect costs, such as patient time spent seeking care. These cost estimates can be applied to cervical cancer-related economic evaluations and support decision-making in Kenya and similar urban settings in other low-income countries.

## Supporting information

S1 Table Calculation methods for per-visit overhead costs for treatment of pre-cancerous lesions and cervical cancer. (DOCX)

S2 Table Percent change in total societal cost of service for pre-cancerous lesion treatment when each individual parameter was varied by +/- 20% via one-way sensitivity analysis under non-integrated, semi-integrated and fully integrated scenarios. (DOCX)

S3 Table Base case assumptions for staff and patient time required for components of cervical cancer treatment. (DOCX)

S4 Table Scenario analyses evaluated for alternative cervical cancer treatment strategies (2017 USD). (DOCX)

S1 File Supporting data. (XLSX)
