# Supplementary material for: Estimating the costs of HIV clinic integrated versus non-integrated treatment of pre-cancerous cervical lesions and costs of cervical cancer treatment in Kenya
Source: PLoS One. 2019 Jun 6;14(6):e0217331. doi: 10.1371/journal.pone.0217331 (PMC6553698; doi:10.1371/journal.pone.0217331)
Supplement: S1 Table — (DOCX) [file pone.0217331.s001.docx]

| **Treatment of** | **Numerator** | **Denominator** | | |
| --- | --- | --- | --- | --- |
| **Pre-Cancerous Lesions** | **All Scenarios** | **Fully Integrated** | **Semi-Integrated** | **Non-Integrated Scenario** |
| Cryotherapy | Total overhead cost estimate from CHC | # of visits (any service) annually | # of visits (any service) annually | # of visits specific to cervical cancer screening and treatment of precancerous lesions |
| Colposcopy | Total overhead cost estimate from CHC | # of visits (any service) annually | 1^st^ visit (integrated): # of visits (any service) annually  2^nd^ visit (non-integrated): # of visits specific to cervical cancer screening and treatment of precancerous lesions | # of visits specific to cervical cancer screening and treatment of precancerous lesions |
| LEEP | Total overhead cost estimate from CHC | # of visits (any service) annually | 1^st^ visit (integrated): # of visits (any service) annually  2^nd^ visit (non-integrated): # of visits specific to cervical cancer screening and treatment of precancerous lesions | # of visits specific to cervical cancer screening and treatment of precancerous lesions |
| **Treatment of** | **Numerator** | **Denominator** | | |
| **Cervical Cancer** |  | **Non-Integrated Scenario Only** | | |
| Adverse Events | Total KNH overhead costs | # of estimated annual visits to KNH  Note: A per-visit overhead cost was calculated. For each care component, the per visit overhead cost was multiplied by the expected number of visits/days for full course of treatment. | | |
| Serious Adverse Events |  |  |  |  |
| Local Invasive Cancer |  |  |  |  |
| Regional Invasive Cancer |  |  |  |  |
| Distant Invasive Cancer |  |  |  |  |
| Follow-Up |  |  |  |  |
| Palliative Care |  |  |  |  |

**S1 Table. Calculation Methods for Per-Visit Overhead Costs for Treatment of Pre-Cancerous Lesions and Cervical Cancer**
