# Supplementary material for: Estimating the costs of HIV clinic integrated versus non-integrated treatment of pre-cancerous cervical lesions and costs of cervical cancer treatment in Kenya
Source: PLoS One. 2019 Jun 6;14(6):e0217331. doi: 10.1371/journal.pone.0217331 (PMC6553698; doi:10.1371/journal.pone.0217331)
Supplement: S2 Table — (DOCX) [file pone.0217331.s002.docx]

**S2 Table. Percent change in total societal cost of service for pre-cancerous lesion treatment when each individual parameter was varied by +/- 20% via one-way sensitivity analysis under non-integrated, semi-integrated and fully integrated scenarios**

|  |  |  |  |  |  |  |  |  |  |  |  |  |  |  |  |
| --- | --- | --- | --- | --- | --- | --- | --- | --- | --- | --- | --- | --- | --- | --- | --- |
| **Parameters Varied +/- 20%** | **Total Cost of Treatment Strategy under Non-Integrated Scenario** | | | | | **Total Cost of Treatment Strategy under Semi-Integrated Scenario** | | | | | **Total Cost of Treatment Strategy under Fully Integrated Scenario** | | | | |
|  | **Base Case** | **-20%** | **+20%** | **% Δ**  **(low)** | **% Δ**  **(high)** | **Base Case** | **-20%** | **+20%** | **% Δ**  **(low)** | **% Δ**  **(high)** | **Base Case** | **-20%** | **+20%** | **% Δ**  **(low)** | **% Δ**  **(high)** |
| *Colposcopy* | | | | | | | | | | | | | | | |
| Direct medical costs for clinical procedure | $91 | $84 | $98 | -7% | 7% | $66 | $59 | $72 | -10% | 10% | $41 | $34 | $48 | -16% | 16% |
| Lab costs | $91 | $89 | $92 | -2% | 2% | $66 | $64 | $67 | -2% | 2% | $41 | $40 | $42 | -3% | 3% |
| Overhead | $91 | $90 | $92 | -1% | 1% | $66 | $65 | $66 | -1% | 1% | $41 | $41 | $41 | 0% | 0% |
| Patient transport | $91 | $85 | $96 | -6% | 6% | $66 | $63 | $68 | -4% | 4% | $41 | $41 | $41 | 0% | 0% |
| Meals for patient and visitors | $91 | $90 | $91 | 0% | 0% | $66 | $66 | $66 | 0% | 0% | $41 | $41 | $41 | 0% | 0% |
| Child/elderly care | $91 | $90 | $91 | -1% | 1% | $66 | $65 | $66 | 0% | 0% | $41 | $41 | $41 | 0% | 0% |
| Productivity loss | $91 | $88 | $93 | -3% | 3% | $66 | $64 | $67 | -2% | 2% | $41 | $41 | $41 | 0% | 0% |
| *Cryotherapy* | | | | | | | | | | | | | | | |
| Direct medical costs for clinical procedure | $46 | $46 | $46 | 0% | 0% | $22 | $17 | $26 | -20% | 20% | $22 | $17 | $26 | -20% | 20% |
| Lab costs | $46 | $46 | $46 | 0% | 0% | $22 | $22 | $22 | 0% | 0% | $22 | $22 | $22 | 0% | 0% |
| Overhead | $46 | $46 | $47 | -1% | 1% | $22 | $22 | $22 | 0% | 0% | $22 | $22 | $22 | 0% | 0% |
| Patient transport | $46 | $44 | $49 | -6% | 6% | $22 | $22 | $22 | 0% | 0% | $22 | $22 | $22 | 0% | 0% |
| Meals for patient and visitors | $46 | $46 | $47 | 0% | 0% | $22 | $22 | $22 | 0% | 0% | $22 | $22 | $22 | 0% | 0% |
| Child/elderly care | $46 | $46 | $47 | -1% | 1% | $22 | $22 | $22 | 0% | 0% | $22 | $22 | $22 | 0% | 0% |
| Productivity loss | $46 | $45 | $48 | -3% | 3% | $22 | $22 | $22 | 0% | 0% | $22 | $22 | $22 | 0% | 0% |
| *LEEP* | | | | | | | | | | | | | | | |
| Direct medical costs for clinical procedure | $99 | $99 | $99 | 0% | 0% | $74 | $65 | $84 | -13% | 13% | $50 | $40 | $60 | -20% | 20% |
| Lab costs | $99 | $99 | $99 | 0% | 0% | $74 | $74 | $74 | 0% | 0% | $50 | $50 | $50 | 0% | 0% |
| Overhead | $99 | $98 | $100 | -1% | 1% | $74 | $74 | $75 | -1% | 1% | $50 | $50 | $50 | 0% | 0% |
| Patient transport | $99 | $94 | $105 | -5% | 5% | $74 | $72 | $77 | -4% | 4% | $50 | $50 | $50 | 0% | 0% |
| Meals for patient and visitors | $99 | $99 | $100 | 0% | 0% | $74 | $74 | $75 | 0% | 0% | $50 | $50 | $50 | 0% | 0% |
| Child/elderly care | $99 | $99 | $100 | -1% | 1% | $74 | $74 | $75 | 0% | 0% | $50 | $50 | $50 | 0% | 0% |
| Productivity loss | $99 | $97 | $102 | -3% | 3% | $74 | $73 | $76 | -2% | 2% | $50 | $50 | $50 | 0% | 0% |
