# Supplementary material for: Estimating the costs of HIV clinic integrated versus non-integrated treatment of pre-cancerous cervical lesions and costs of cervical cancer treatment in Kenya
Source: PLoS One. 2019 Jun 6;14(6):e0217331. doi: 10.1371/journal.pone.0217331 (PMC6553698; doi:10.1371/journal.pone.0217331)
Supplement: S3 Table — (DOCX) [file pone.0217331.s003.docx]

**S3 Table. Base Case Assumptions for Staff and Patient Time Required for Components of Cervical Cancer Treatment**

|  | **Time Required** |
| --- | --- |
| Radiotherapy:  (28 sessions total for full course) | Low: 15 minutes per session  Average: 37.5 minutes High: 60 minutes |
| Chemotherapy: (3 sessions total for full course) | Low: 30 minutes per session Average: 2 hours per session High: 4 hours per session |
| Palliative Care  (one facility-based visit) | Low: 48 minutes per session Average: 1 hour per session High: 1.5 hours per session |

*Staff and patient time generally required (on average) to provide radiotherapy and chemotherapy to cervical cancer patients. These time estimates will be incorporated into costs of providing these services to patients (Staff Time x Wage; Patient Time x Wages Missed).*
