# Supplementary material for: Estimating the costs of HIV clinic integrated versus non-integrated treatment of pre-cancerous cervical lesions and costs of cervical cancer treatment in Kenya
Source: PLoS One. 2019 Jun 6;14(6):e0217331. doi: 10.1371/journal.pone.0217331 (PMC6553698; doi:10.1371/journal.pone.0217331)
Supplement: S4 Table — (DOCX) [file pone.0217331.s004.docx]

**S4 Table. Scenario Analyses Evaluated for Alternative Cervical Cancer Treatment Strategies (2017 USD)**

|  |  |  |  |
| --- | --- | --- | --- |
| **Severity** | **Stage** | **Treatment Option*** | **Direct Medical Costs**** |
| Local Invasive | IA1; IA2; IB1; IIA1 | Radical Hysterectomy/Surgery | $1,750.07 |
|  |  | Simple Hysterectomy/Surgery | $1,276.59 |
|  |  | Radiotherapy  (Contraindicated for Surgery) | $4,366.09 |
|  |  | Simple Hysterectomy + Radiotherapy | $5,253.87 |
| Regional Invasive | IB2; IIA2; IIIA; IIIB | Radical Hysterectomy + Chemotherapy + Radiotherapy | $6,437.59 |
|  |  | Chemotherapy + Radiotherapy | $5,076.32 |
|  |  | Radiotherapy | $4,366.09 |
| Distant Invasive | IVA; IVB | Chemotherapy + Radiotherapy + Palliative Care | $5,113.01 |
|  |  | Chemotherapy + Radiotherapy | $5,076.32 |
|  |  | Palliative Care (Single Visit) | $36.70 |

**Radiotherapy costs include 28 sessions. Chemotherapy costs include 3 sessions. Palliative Care include a palliation consult, family therapy, wound dressing, renewal of prescriptions, and rehydration services.*

***Direct medical costs presented here for hysterectomy, radiotherapy and chemotherapy represent the procedure fee for each type or combination of treatment services, plus the direct medical costs for (1) Staging and labs: $105.38; (2) Consultation: $6.51; (3) Medications for pain relief: $118.37.*
